# Improving Patient Satisfaction and Reducing Agitation in Overcrowded Outpatient Waiting Areas Through Environmental and Educational Audiovisual Interventions: A Single-Centre Quality Improvement Project

**DOI:** 10.7759/cureus.102349

**Published:** 2026-01-26

**Authors:** Manu D Kandachamkulam, Merin A Job, Eby D Kandachamkulam, Meera S N

**Affiliations:** 1 Internal Medicine, Government Medical College and Hospital, Thiruvananthapuram, Thiruvananthapuram, IND; 2 Internal Medicine, AJ Hospital, Thiruvananthapuram, IND; 3 Emergency Medicine, Government Medical College and Hospital, Thiruvananthapuram, Thiruvananthapuram, IND

**Keywords:** agitation, audiovisual intervention, environmental design, outpatient department, patient experience, plan–do–study–act, quality improvement, single-centre study, verbal altercations, waiting time

## Abstract

Background: Overcrowding and prolonged waiting times in outpatient departments (OPDs) are frequently associated with patient dissatisfaction, agitation, and conflict, particularly in high-volume public healthcare settings. Structural and workforce constraints often limit the feasibility of reducing waiting times in the short term, necessitating alternative strategies to improve patient experience and reduce agitation. This study was conducted as a quality improvement initiative aimed at enhancing patient experience in a high-volume public sector outpatient setting.

Methods: We conducted a single-centre quality improvement (QI) initiative using a Plan-Do-Study-Act (PDSA) framework over an eight-week period in a high-volume Internal Medicine OPD of a tertiary care public hospital in South India. Weeks 1-4 constituted the pre-intervention phase and Weeks 5-8 the post-intervention phase. The intervention involved the installation of televisions in OPD waiting areas displaying curated, non-disruptive, family-friendly audiovisual content along with short health awareness videos in the local language. All patients attending the OPD during the study period were included, with the sample size determined by consecutive attendance. Weekly OPD attendance and reported verbal altercations were recorded. Altercation rates were calculated per 1,000 OPD visits, and pre- and post-intervention weekly means were compared using descriptive statistics and inferential analysis.

Results: Mean weekly OPD attendance remained comparable between the pre- and post-intervention periods (2,228 vs. 2,253 patients/week). Mean reported verbal altercations decreased significantly from 39 per week during the pre-intervention phase to 7.25 per week post-intervention, representing an approximate 81% reduction (p < 0.01). When adjusted for patient volume, altercation rates declined from approximately 17.5 to 3.2 per 1,000 OPD visits. The reduction was sustained across all weekdays, including traditionally high-volume days. Average waiting times remained unchanged.

Conclusion: A low-cost environmental and educational audiovisual intervention was associated with a substantial and sustained reduction in reported patient agitation without altering patient throughput or waiting times. Simple, scalable quality improvement strategies that enhance the waiting-area experience may meaningfully improve patient-centred care in resource-constrained outpatient settings.

## Introduction

High patient volumes and prolonged waiting times are common challenges in public sector outpatient departments and are frequently associated with patient dissatisfaction, agitation, and conflict. In our institution, recurrent verbal altercations between patients, bystanders, and healthcare staff posed concerns regarding patient safety, staff morale, and workflow efficiency. System-level constraints precluded immediate reductions in waiting times or staffing augmentation. This quality improvement project aimed to evaluate whether a low-cost environmental and educational audiovisual intervention could reduce patient agitation and conflict despite persistent crowding.

Patient experience in outpatient settings is influenced not only by actual waiting times but also by environmental and contextual factors that shape perceived waiting experience [1,2]. Prior studies have demonstrated that environmental modifications, such as audiovisual distraction, ambient design, and purposeful engagement, may reduce anxiety, improve satisfaction, and positively influence patient behaviour in healthcare environments [2]. Systematic reviews of healthcare environmental design have reported associations with improved patient outcomes, including reduced stress and enhanced satisfaction [1].

Such interventions are particularly appealing in high-volume, resource-limited settings where structural expansion or workforce increases are not feasible. Incorporating basic health education content into waiting-area environments may further enhance patient engagement by transforming idle waiting time into a more meaningful experience. However, evidence from public sector OPDs in low- and middle-income countries remains limited. This project sought to assess the impact of a simple, scalable environmental intervention within such a context.

## Materials and methods

Design and setting

This single-centre quality improvement project was conducted in the Internal Medicine OPD of a tertiary care public hospital in South India using a Plan-Do-Study-Act (PDSA) methodology and was reported in accordance with SQUIRE 2.0 guidelines on quality improvement practice [3].

The project was conducted in the Internal Medicine OPD of Government Medical College Hospital, Thiruvananthapuram, a tertiary care center with a high daily outpatient volume. The OPD caters to approximately 300-400 patients per clinic day. All patients and accompanying attendants present in the waiting areas during the study period were included, reflecting real-world OPD conditions.

A substantial proportion of patients attending this OPD had already undergone initial assessment and stabilization in the emergency department (casualty) before being referred for outpatient physician evaluation, often contributing to prolonged waiting times exceeding four hours.

Intervention

The audiovisual content consisted of two complementary streams with predefined duration and structure. The first stream included family-friendly entertainment material such as non-violent films, short comedy segments, music programmes, and nature videos, each typically 10-20 minutes in length. All content was pre-screened by the project team to ensure cultural appropriateness, absence of distressing themes, and suitability for a mixed-age audience. No commercial advertisements, sponsorship messages, or promotional material were displayed at any time.

The second stream comprised structured health education videos, each 2-5 minutes in duration, addressing common conditions encountered in the clinic, including diabetes mellitus, hypertension, medication adherence, hand hygiene, and prevention of locally prevalent infectious diseases. These videos were delivered in the local language using simple narration, pictorial demonstrations, and minimal medical jargon to ensure comprehension across varying literacy levels.

Entertainment and educational segments were alternated in a continuous loop throughout OPD hours, maintaining an approximate 3:1 ratio of entertainment to educational content. The playlist remained fixed for the duration of each week to ensure uniform exposure for all attendees. New playlists were introduced at the start of every subsequent week, as the department functions through six rotating clinical units and most patients do not re-attend the OPD more than once within the same week. Audio volume was maintained at a moderate, non-intrusive level so as not to interfere with clinical communication. The overall intent was to provide passive distraction while simultaneously utilising waiting time for brief, low-intensity health promotion without exposing patients to any commercial influence.

Data collection and study period

Baseline data were collected over four weeks prior to intervention implementation. Post-intervention data were collected over another four-week period following the installation of the televisions. Data were collected weekly and included: (1) total OPD attendance. (2) Number of reported verbal altercations documented in routine incident logs. Each altercation was recorded using a standardized incident log, including date, time, location, personnel involved, and a brief description of the incident. Logs were reviewed weekly by the project team for accuracy and completeness. (3) Informal staff and patient feedback regarding the waiting-area environment. All OPD staff involved in patient care received brief training on completing incident reports consistently and objectively, emphasizing timely documentation and maintaining confidentiality of patients and staff.

Sample size considerations 

All patients attending the Internal Medicine OPD during the study period were included. A formal sample size calculation was not performed, as this was a pragmatic quality improvement initiative aimed at evaluating the impact of a service-level intervention under real-world conditions. The sample size was determined by consecutive OPD attendance over the defined study period, consistent with standard QI methodology.

Statistical analysis

Descriptive statistics were used to summarise weekly OPD attendance and altercation counts. Altercation rates were calculated per 1,000 OPD visits to account for variations in patient volume. Data were analysed using Microsoft Excel 2019 (Microsoft Corp., Redmond, WA, USA). A post hoc chi-square test was performed using Microsoft Excel 2019 (Microsoft Corp., USA) to compare observed altercation counts in the pre- and post-intervention periods against an expected equal distribution. This analysis assessed deviation from equal distribution only and does not establish causality between the intervention and outcome.

Ethical considerations

This project was conducted as a quality improvement initiative aimed at service enhancement. No patient-identifiable data were collected. Formal institutional ethics committee approval was not required as per local policy.

Patient consent

Informed consent was waived as this study involved anonymised, aggregated operational data collected as part of routine quality improvement activities.

PDSA cycle description

Plan

Key contributors to agitation were identified, including prolonged idle waiting, boredom, and environmental stressors. A feasible, low-cost environmental intervention was selected, with a target of reducing altercations by at least 50% over eight weeks. The content strategy deliberately combined passive distraction with basic health education to improve engagement during prolonged waiting periods.

Do

Televisions were installed in high-density waiting areas, and curated audiovisual content was implemented without altering routine OPD operations. Content governance procedures and a weekly rotation protocol were instituted to ensure the appropriateness of material, consistency of delivery, and avoidance of repetition for patients attending different clinic units. All OPD staff received brief training on completing incident reports consistently and objectively, emphasizing timely documentation and confidentiality.

Study

Weekly OPD attendance and altercation data were reviewed. Verbal altercations were documented using standardized incident logs including date, time, location, personnel involved, and a brief description. Logs were reviewed weekly by the project team for accuracy and completeness. Informal feedback from staff and patients was gathered.

Act

The intervention was retained permanently, with recommendations for expansion to other outpatient departments and potential incorporation of additional patient-education content in future cycles.

## Results

**Figure 1:**
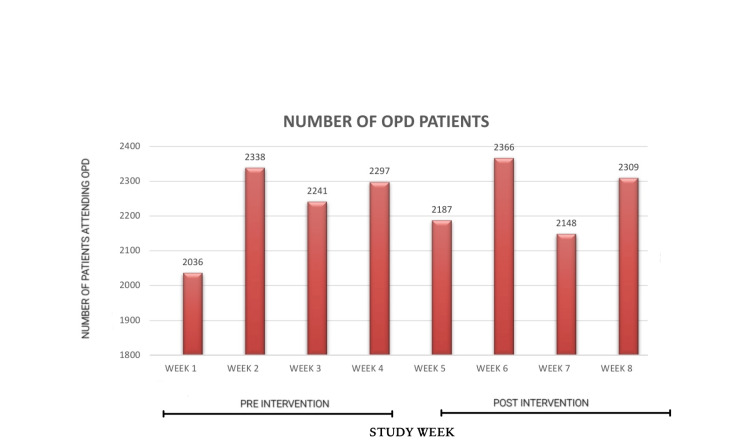
Total number of patients attending OPD. OPD: outpatient department.

**Figure 2:**
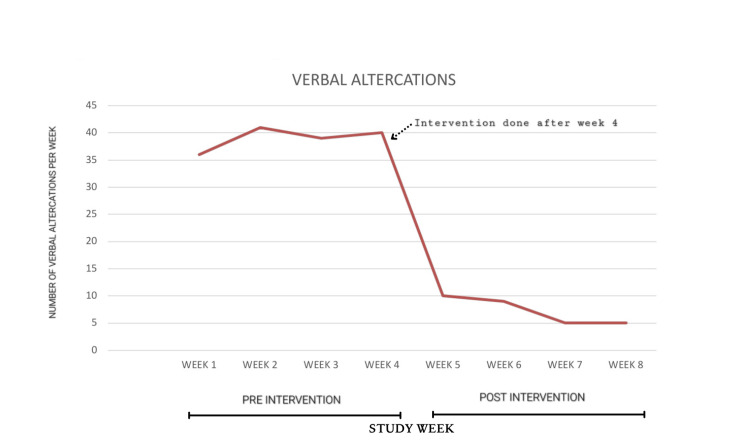
Number of verbal altercations in OPD. OPD: outpatient department.

Weekly OPD attendance remained stable across the study period (Figure 1). A marked and sustained reduction in reported verbal altercations was observed following implementation of the audiovisual intervention (Figure 2). The audiovisual playlist and weekly rotation protocol were implemented with full adherence throughout the post-intervention phase, and no interruptions in content delivery were recorded.

Mean weekly altercations decreased from 39 during the pre-intervention period to 7.25 during the post-intervention period, representing an approximately 81% reduction in reported altercations. When adjusted for OPD volume, altercation rates decreased from approximately 17.5 to 3.2 per 1,000 patient visits. In addition, a post hoc chi-square analysis comparing observed altercation counts across the pre- and post-intervention periods against an expected equal distribution demonstrated a statistically significant reduction in altercations following the intervention (p < 0.01).

Altercations were most frequent on Mondays and Saturdays during the pre-intervention phase, corresponding to higher patient volumes. Following the intervention, altercation frequency decreased substantially even on these traditionally high-volume days. Patients experiencing prolonged waiting times exceeding four hours did not demonstrate a proportional increase in altercations.

## Discussion

This quality improvement project demonstrates that a simple, low-cost environmental modification, the installation of televisions displaying calming, family-friendly content, was associated with a meaningful reduction in reported patient agitation and improved satisfaction in a high-volume outpatient department (OPD), despite persistently prolonged waiting times. Although average waiting times remained unchanged, the marked decline in verbal altercations suggests that patients’ subjective experience of waiting may exert a greater influence on agitation than the absolute duration of the wait.

Comparison with existing literature

Our findings align with existing evidence indicating that the physical and sensory environment of waiting areas impacts patient satisfaction and behavior. Laursen et al. conducted a systematic review showing that environmental design in healthcare settings, including aspects like noise, lighting, and amenities, can influence patient outcomes including anxiety and satisfaction [1] . Similarly, MacAllister et al. identified specific environmental variables, such as comfort, sensory stimuli, and spatial layout, that are associated with higher levels of patient satisfaction [2]. These studies support the premise that improving the waiting environment can positively influence patient perceptions and reduce stress even in the absence of reduced wait times. Further supporting this, Tsai et al. found that elements of the physical environment, such as visual and auditory conditions, are significantly associated with outpatient perceptions of the waiting experience. Patients’ satisfaction scores across dimensions like lighting, noise levels, seating comfort, and cleanliness varied with individual characteristics, suggesting that an improved environment contributes to overall satisfaction beyond structural variables alone [4]. While Tsai’s study did not focus on audiovisual distractions like televisions, it underscores the broader role of environmental conditions in shaping waiting area experiences [4].

Notably, in our project, patients with waiting periods exceeding four hours, many of whom had been evaluated in casualty prior to being referred to the OPD, also exhibited reduced agitation post-intervention. This observation aligns with evidence suggesting that environmental distractions, such as audiovisual content or reading materials, can mitigate the negative psychological impact of prolonged waiting. For example, studies of outpatient clinics have found that increasing the availability of distractions like television or magazines is associated with enhanced satisfaction and reduced negative affect among waiting patients.

Interpretation of findings

The significant reduction in verbal altercations despite unchanged waiting times highlights the importance of perception in shaping patient behaviour. Individuals experiencing long waits may feel less frustrated when the environment offers engaging or soothing stimuli that divert attention from idle waiting. This finding reinforces the psychological principle that perceived time is influenced by engagement rather than by elapsed time alone, a concept well established in the patient-experience literature [4]. Although waiting time remains an important determinant of satisfaction, patients’ comfort, level of engagement, and sense of support during the wait appear to play equally critical roles.

Strengths and limitations

Key strengths of this project include the pragmatic nature of the intervention, its minimal cost, and its implementation without disruption to existing clinical workflows. The use of a Plan-Do-Study-Act cycle enabled structured, iterative learning within a real-world clinical environment. Limitations include the single-centre design, reliance on routinely collected incident logs and informal feedback rather than validated psychometric instruments, and a relatively short follow-up period. Reported altercations were based on routine documentation and may therefore be subject to under-reporting and observer variability. Additionally, the project focused primarily on quantitative outcomes; incorporation of structured qualitative assessments in future work could provide deeper insight into how and why environmental modifications influence patient behaviour.

Implications for practice and future research

These findings suggest that straightforward, low-cost environmental modifications can meaningfully enhance patient experience in high-volume outpatient settings, even when systemic constraints limit opportunities to reduce waiting times. Audiovisual interventions in waiting areas may represent a pragmatic and scalable strategy for healthcare facilities seeking to decrease patient agitation and improve staff-patient interactions without altering clinical processes or resource allocation. The intervention required minimal maintenance expenditure and was integrated into routine operations, supporting its feasibility for long-term sustainability.

From a practical standpoint, such approaches can be readily adopted in resource-constrained public hospitals and tailored to local cultural and linguistic contexts. Combining passive distraction with basic health education content may further enhance engagement by transforming idle waiting into a more purposeful and reassuring experience. These strategies may be particularly valuable in settings characterised by prolonged waits, high patient turnover, and limited infrastructure for crowd management.

Although the reduction in agitation observed in this project was substantial, the work represents an initial quality-improvement cycle conducted over a limited period in a single institution. Future studies should incorporate longer follow-up, repeated PDSA cycles, and multicentre implementation to evaluate sustainability, generalisability, and contextual determinants of effectiveness. The use of validated patient-experience instruments would permit more robust measurement of subjective outcomes and facilitate comparison across settings.

In addition to structured questionnaires, future research could include open-ended patient narratives to capture nuanced perceptions of the waiting environment. Qualitative analytic approaches, such as thematic analysis or text-mining techniques, may yield richer understanding of emotional responses, perceived stressors, and the aspects of the intervention most valued by patients. Further work should also explore the comparative effectiveness of different forms of audiovisual content, the impact of combined environmental strategies (for example, calming music or reading materials), and longer-term outcomes including patient satisfaction scores, repeat attendance, and staff-reported conflict.

## Conclusions

In a high-volume outpatient department with persistent crowding, a low-cost environmental and educational audiovisual intervention was associated with a substantial and sustained reduction in reported patient agitation and conflict without affecting patient throughput. Pragmatic quality improvement strategies that enhance the waiting-area experience may offer meaningful improvements in patient-centred care in resource-constrained healthcare settings.
